# Red Deer as Maintenance Host for Bovine Tuberculosis, Alpine Region

**DOI:** 10.3201/eid2103.141119

**Published:** 2015-03

**Authors:** Maria Fink, Corina Schleicher, Monika Gonano, Wolfgang M. Prodinger, Maria Pacciarini, Walter Glawischnig, Marie-Pierre Ryser-Degiorgis, Chris Walzer, Gabrielle L. Stalder, Dorotea Lombardo, Hermann Schobesberger, Petra Winter, Mathias Büttner

**Affiliations:** Austrian Agency for Health and Food Safety, Moedling, Austria (M. Fink, M. Gonano);; Austrian Agency for Health and Food Safety, Graz, Austria (C. Schleicher);; Innsbruck Medical University, Innsbruck, Austria (W.M. Prodinger);; Istituto Zooprofilattico Sperimentale della Lombardia e dell’Emilia Romagna, Brescia, Italy (M. Pacciarini);; Austrian Agency for Health and Food Safety, Innsbruck (W. Glawischnig);; University of Bern, Bern, Switzerland (M.-P. Ryser-Degiorgis);; University of Veterinary Medicine, Vienna, Austria (C. Walzer, G.L. Stalder, H. Schobesberger, P. Winter);; Istituto Zooprofilattico Sperimentale delle Venezie, Bolzano, Italy (D. Lombardo);; Bavarian Health and Food Safety Authority, Oberschleißheim, Germany (M. Büttner)

**Keywords:** Tuberculosis and other mycobacteria, wildlife, bovine tuberculosis, Mycobacterium caprae, reservoir host, spillover host, spillback host, maintenance host, red deer, Alps, zoonoses, bacteria

## Abstract

To estimate the prevalence of bovine tuberculosis in the Alpine region, we studied the epidemiology of *Mycobacterium caprae* in wildlife during the 2009–2012 hunting seasons. Free-ranging red deer (*Cervus elaphus*) were a maintenance host in a hot-spot area, mainly located in Austria.

Bovine tuberculosis has one of the broadest host ranges of any known zoonotic pathogens. In addition to cattle, bovine tuberculosis affects many wild animal populations in North America, Europe, Africa, Asia, and New Zealand. Under certain conditions, wildlife play a role as reservoir and source of infection for domestic animals. *Mycobacterium caprae* has been isolated from cattle, domestic goats, domestic pigs, red deer (*Cervus elaphus*), and wild boar (*1*). Evidence is increasing that *M. caprae* is emerging in free-ranging red deer and cattle in the Alps (*2*,*3*).

## The Study

To estimate the prevalence of bovine tuberculosis (which is caused by *M. bovis* and *M. caprae*) in wildlife in the Alps, we investigated 1,655 hunted red deer of both sexes and different ages in Austria, Germany, Switzerland (including the Principality of Liechtenstein), and Italy. The deer were hunted specifically for the study by trained hunters. A sampling/hunting plan was calculated in advance that indicated the number of animals needed in each sampling region to calculate prevalence estimates; the number was based on the red deer density of a region. The numbers of animals killed and sampled during 3 consecutive hunting seasons (2009–10, 2010–11, and 2011–12) coordinated nearly perfectly with the sampling plan that had been developed for each sampling area (Table 1). After pathomorphologic examination of carcasses (from Germany, Austria, Italy, Swiss Tessin) or samples (from Swiss St. Gall, Swiss Grisons, and Liechtenstein), we conducted microbiological analysis from sample material. Sample material included both medial retropharyngeal lymph nodes and tracheobronchial, mediastinal, and mesenteric lymph nodes and any other tissue with macroscopically visible lesions (Table 2). For bacteriologic cultivation the sample material was homogenized by using the IKA Ultra Turaxx Tube Drive System (Staufen, Germany), decontaminated with 1% N-acetyl-L-cystein solution and neutralized in phosphate buffer (pH 6.8) as recommended by the World Organisation for Animal Health (*4*). After sedimentation, inoculation was performed on 2 growth media: Stonebrink including PACT (polymyxin B, amphotericin B, carbenicillin, and trimethoprim) and Lowenstein-Jensen with glycerin and PACT (Heipha Diagnostika, Eppelheim, Germany). After 12 weeks’ incubation, a total of 82 bacterial cultures from 59 hunted red deer from Austria, Germany, and Italy were isolated (Tables 1, 2). All isolates were identified as *M. caprae* whether by reversed line blotting (Geno Type MTBC, HAIN Lifescience, Nehren, Germany) or by restriction fragment length polymorphism PCR of the *gyrB* gene, as previously described (*5*).

**Table 1:** Estimated prevalence of *Mycobacterium caprae* in red deer (*Cervus elaphus*), Alpine region, 2009–10, 2010–11, and 2011–12 hunting seasons

Study area	No. animals	No. *M. caprae* positive	Estimated prevalence (95% CI), %
Austria, total	590	55	
Tyrol			
Lechtal I	173	40	23.1 (17.0–30.2)
Lechtal Mitte	98	7	7.1 (2.9–14.2)
Lechtal II	15	1	6.7 (0.1–32.0)
Tannheimertal	32	0	0 (0.0–9.0)
Schwarzwasser	38	0	0 (0.0–7.6)
Vorarlberg			
Region 1	50	0	0 (0.0–5.9)
Region 2	61	1	1.6 (0.0–8.8)
Region 3	47	6	12.8 (4.8–25.8)
Region 4	41	0	0 (0.0–7.1)
Region 5	35	0	0 (0.0–8.3)
Switzerland, total	273	0	
Grisons	88	0	0 (0.0–3.4)
St. Gall	48	0	0 (0.0–6.1)
Tessin	89	0	0 (0.0–3.4)
Liechtenstein	48	0	0 (0.0–6.1)
Italy, total	514	1	
Bergamo/Brescia	77	1	1.3 (0.0–7.1)
Bolzano			
East	23	0	0 (0.0–12.3)
North	29	0	0 (0.0–9.9)
South	10	0	0 (0.0–25.9)
West	60	0	0 (0.0–4.9)
Como/Lecco	61	0	0 (0.0–4.8)
Sondrio	95	0	0 (0.0–3.2)
Trento			
East	41	0	0 (0.0–7.1)
West	53	0	0 (0.0–5.5)
Varese	65	0	0 (0.0–4.6)
Germany, total	278	3	
Region 1	187	1	0.5 (0.0–3.0)
Region 2	91	2	2.2 (0.2–7.8)

**Table 2:** *Mycobacterium caprae*–positive red deer (*Cervus elaphus*) and occurrence of macroscopically visible lesions in selected lymph nodes and other tissues, Alpine region, 2009–10, 2010–11, and 2011–12 hunting seasons*

No. animals, N = 59	Lymph node†				Other tissue‡
	Retropharyngeal	Mediastinal	Tracheobronchial	Mesenteric	
29	+	NLD	NLD	NLD	NLD
5	NLD	NLD	NLD	+	NLD
4	NLD	NLD	NLD	NLD	NLD
3	+	NLD	NLD	+	NLD
2	NLD	NLD	+	NLD	Lung
1	+	NLD	+	NLD	Lung
1	+	NLD	NLD	+	Tonsil
1	NLD	NLD	NLD	NLD	Parotid lymph node
2	+	+	+	NLD	NLD
2	NLD	NLD	+	+	NLD
1	+	+	+	+	NLD
1	NLD	+	+	+	NLD
1	+	NLD	+	+	NLD
1	+	+	NLD	+	NLD
1	NLD	+	NLD	+	NLD
1	+	NLD	+	NLD	NLD
1	+	+	NLD	NLD	NLD
1	NLD	NLD	+	NLD	NLD
1	NLD	+	NLD	NLD	NLD

*NLD, no lesions detected; +, macroscopically visible lesions. †No. animals with positive findings: retropharyngeal, 41 deer; mediastinal, 8 deer; tracheobronchial, 12 deer; mesenteric, 16 deer. ‡No. animals with positive findings: 5 deer.

Red deer with macroscopically visible lesions (purulent abscesses varying remarkably in size) were all *M. caprae* positive from >1 lymph nodes/tissue, and 4 animals without visible lesions were *M. caprae* positive (Table 2). Microscopic examination showed that lesions had thin-walled fibrous capsules, occasionally with neutrophil granulocytes and calcifications in the necrosis zone, with an increased occurrence of neutrophil granulocytes and calcifications around the necrosis zone, and with a high occurrence of epithelioid and giant cells at the inside capsule wall. The large majority of these lesions were in the lymph nodes, particularly the medial retropharyngeal lymph nodes. Lesions in the lungs, observed in 3 animals, were consistently found in combination with lesions in the tracheobronchial and mediastinal lymph nodes; lesions in the tonsils were found with lesions in the retropharyngeal lymph nodes (Table 2). The medial retropharyngeal lymph nodes and the mesenteric lymph nodes were the primary sites of infection; most of the deer had lesions in the medial retropharyngeal lymph nodes and in the mesenteric lymph nodes, either exclusively or in combination with other sites (Table 2). Furthermore, the 4 animals without visible lesions were *M. caprae* positive from the medial retropharyngeal lymph nodes, suggesting a very early stage of infection.

*M. bovis* infections of cattle or deer tonsils led to bacterial colonization in the medial retropharyngeal lymph nodes with subsequent lymphatic spread to pulmonary lymph nodes. Ingestion of the *M. bovis* bacteria led to lymphatic spread from the primary infection, the gut, to mediastinal or tracheobronchial lymph nodes, which explained the lesions in the mesenteric lymph nodes (*6*). *M. bovis* causing tuberculosis is proposed to be a lymphatic disease (*6*); we propose the same for *M. caprae*.

**Figure 1:**
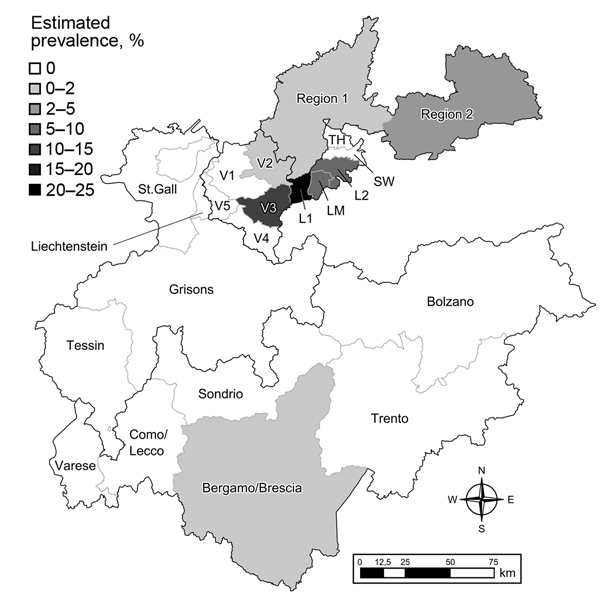
Study area in the Alpine region showing the 22 sampling regions and the estimated prevalences of *Mycobacteria caprae*. Prevalence ranges are classified into 6 intervals, wherein the upper bounds are not included in the interval. Austria: Vorarlberg (V1–V5) and Tyrolean Lech valley: Lechtal I (L1), Lechtal Mitte (LM), Lechtal II (L2), Schwarzwasser (SW),and Tannheimertal (TH). Germany: Bavaria (Region 1 and Region 2). Switzerland: St. Gall, Grisons, Tessin, and Liechtenstein. Italy: Varese, Como/Lecco, Sondrio, Bergamo/Brescia, Trento, Bolzano.

**Figure 2:**
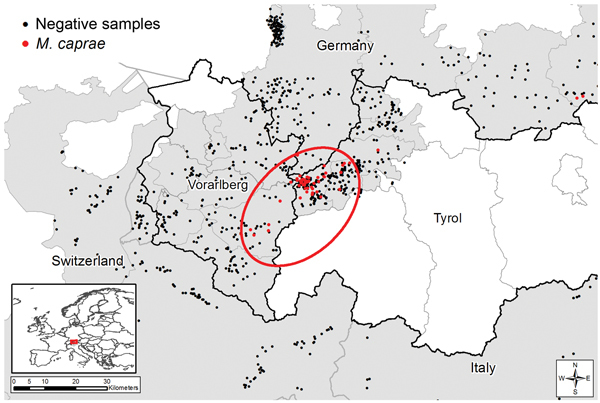
Statistically evident spatial cluster of Mycobacterium caprae–positive red deer in the Alpine region, 2009-10, 2010-2011, and 2011-12 hunting seasons. Area in red circle contained significantly more *M. caprae*–positive red deer than the remaining study area (p<0.001). Inset shows location of Austria and Germany within Europe (shading).

We calculated regional prevalence estimates with 95% CIs (Table 1; Figure 1). Prevalence estimates differed greatly among the sampling areas in Austria, Switzerland, Germany, and Italy. Moreover, a spatial cluster of positive samples (hot spot) around the Austrian sampling area Lechtal I was identified using point pattern analysis. The cluster comprised all positive animals from Lechtal I and the contiguous sampling areas of Lechtal Mitte, Regions 2 and 3 of Vorarlberg, and Region 1 of Bavaria (Germany) (p<0.001) (*7*–*10*). Consequently, this hot spot included 55 of the 59 positive samples detected in this study (Table 1; Figure 2).

Genotyping was performed from *M. caprae* isolates by spoligotyping (*11*) and by mycobacterial interspersed repetitive unit typing and variable number tandem repeat genotyping for 24 loci (*12*). All isolates in the hot-spot area and the 2 other isolates from Austria were of the Lechtal genotype. The single isolate in Italy, located >200 km from the hot spot, was also of the Lechtal genotype, and an association might exist between this case and cattle previously imported from Austria to Italy (*13*). By contrast, 2 isolates from Germany found in the Karwendel Mountains were of the Karwendel genotype. All other deer were negative for *M. caprae* and for *M. bovis*. After the transnational project, in the 2012–13 hunting season, the prevalence in Bavaria 1 (Germany) was 5.3%, indicating an expansion of or shift in the hot spot.

## Conclusions

Our data indicate a localized bovine tuberculosis problem in wildlife in the Alps related to wildlife management strategies. Supplementary feeding is common in the hot-spot area to prevent migration and to keep red deer populations high (*3*). In the hot-spot area, the overall density is 5.6 animals/km^2^, but because of aggregation, which is considered the main cause of bovine tuberculosis maintenance in wildlife, winter habitats of red deer around feeding sites have up to 46.2 animals/km^2^ (Statistik Austria, http://www.statistik.at) (*14*). Low (0.5–2.5 and 2–4/km^2^) or medium densities (9.7 deer/km^2^) with prevalence estimates of 0% were found in Switzerland (Saint Gall, Liechtenstein, and Grisons, respectively) (*14*). Brescia, the only place in Italy where *M. caprae* was found in red deer, is considered a high-density region (16 animals/km^2^), but management strategies promoting aggregation are largely absent in Italy and Switzerland (*13*). Furthermore, red deer in the hot-spot area are a reservoir for bovine tuberculosis and are the source of *M. caprae* infections for domestic cattle grazing on Alpine pastures during the summer (*3*). Molecular-epidemiologic studies indicate that *M. caprae* isolates from red deer and cattle are of the same genotype (*2*). Our finding that retropharyngeal lymph nodes and mesenteric lymph nodes were often the primary site of infection suggests oral rather than aerosol transmission. Oral transmission does not require direct contact, either among red deer or between red deer and cattle.
